# Supplementary material for: Complexome profiling of the Chlamydomonas psb28 mutant reveals TEF5 as an early PSII assembly factor
Source: Plant Cell. 2025 Mar 17;37(6):koaf055. doi: 10.1093/plcell/koaf055 (PMC12164590; doi:10.1093/plcell/koaf055)
Supplement: koaf055_Supplementary_Data [file koaf055_supplementary_data.zip › psb28_interactive.zip!/plots/intensity/qualityPlots/peptides/CCB1_Cre16.g662150.t1.2.html]

### Cre16.g662150.t1.2

Trivial name: CCB1  
SeqCoverage [median]: 24.90  
Mass: 29.61 kDa  
  
 

| Sequence | MissedCleavage | Mass [Da] | wtIntensity | muIntensity | Score |
| --- | --- | --- | --- | --- | --- |
| ELGLVEK | 0 | 786.45 | 1173.14 | 0.00 | 72.14 |
| LTFEVPGPAVEGAMSLDDR | 0 | 2002.97 | 2026.89 | 0.00 | 93.37 |
| LTFEVPGPAVEGAMSLDDRAR | 1 | 2230.11 | 0.00 | 2848.63 | 49.22 |
| MVTATDDQTTDIIVEGDKEEIER | 1 | 2607.22 | 1027.56 | 8714.53 | 137.05 |
| VYVKGMLEA | 1 | 1008.53 | 0.00 | 2311.54 | 106.36 |
| YNYDVK | 0 | 800.37 | 708.93 | 5646.18 | 114.02 |
|  |  |  | 4936.5 | 19520.9 |  |
